# Mother and Child in Scotland

**Published:** 1918-06-15

**Authors:** 


### CARNEGIE REPORTS ON PHYSICAL WELFARE.

### (4) MOTHER AND CHILD IN SCOTLAND.

THE fourth volume of the Carnegie Trust Report on the Physical Welfare of Mothers and Children deals with Scotland, and is again an immense volume, weighty in more senses than one. In view of the great value to thinkers and workers for social betterment it seems a pity that some kind of popular digest of the vast mass of information collected could not be put forth for general reading, enriched by summaries at least of the conclusions come to by the various officials and specialists who carried out the inquiries. As it is, these great tomes will no doubt remain as the best possible *mémoires pour servir* for the future historians of progress. Meantime they mark the stage reached at a time when philanthropic effort is following the course of all other national service in this country, and passing from the hands of volunteer pioneers into those of constituted authority. If the case for a National Ministry of Health required proving, that is accomplished here in the need of co-ordination and re-direction of effort shown.

In a memorable phrase Dr. Leslie Mackenzie begins his report by describing the map of Scotland as his "official praying-machine," daily offering the reminder that within its limits there is nothing indifferent, or to be ignored or neglected. In fact, Scotland presents the familiar problems of England and Wales in an acute form. A similar congestion of population into industrial centres proceeds "at breakneck speed," so that at the present moment the Highlands and Islands form more than half the area of the country, but contain only one-tenth of the population. The problems of motherhood and infancy in the overcrowded city and the lonely cottage are therefore equally forcibly illustrated, and their study is here undertaken in the spirit of that "distinguished administrator" quoted by the Secretary to the Trust, who longed to settle himself in some one neighbourhood and remain there long enough to find out why the children died. That is our national problem: to find out how we can stop the wastage and the damage of young life.

Each investigation pushes the subject further back, and prematernity problems naturally occupy more space in this volume. Statistics are dreary things, but, as Florence Nightingale so often showed, they become instinct with life and purpose when they point the way to a cure for evils. Scotland in any year has 110,000 to 120,000 expectant mothers. In the five years 1911-15 "congenital causes"—prematurity, wasting, debility, malformations—killed 23,481 infants under one year. Roughly, some 4,600 children die in Scotland every year; that is, 46,000 citizens are lost in a decade, and in every variety of community the death-rate is highest in the first week. Dr. Ballantyne's essay on the care of the expectant mother has many points of interest. For instance, he lays stress on the need for more attention to miscarriages and to the infant prematurely born, to save it from adding an item to the death-rate or a weakling to the population. Prevention of prematurity by care of the mother would do much, but care in small wards properly fitted and staffed, and special commendation of the child to the school medical officer (and until that stage, we will hope, attention at a child-welfare centre), would nurse many a feeble child to robust health. We need to grasp the writer's dictum that such a babe is "a fœtus out of his proper environment," so that the wind may be tempered to his delicate frame with success. Moreover, since the placental tissues may give the first indication of venereal infection, better prospects may be assured for a following child. Hitherto the lack of this kind of care has been a weak point in our national system. Naturally, the difficult question of the compulsory or the willing notification of pregnancy is involved. Dr. Mackenzie is of opinion that for years to come the cases voluntarily asking advice at maternity centres will be more numerous than any local authority can manage, and we shall all agree with him that the urgent need is for immediate provision of great numbers of such centres, largely staffed by women doctors. Every social worker is familiar with the fact that the uneducated woman finds a difficulty much greater than that o[illegible] trained mind in confiding her story of suffering t[illegible] man doctor. The testimony of this Report to the supe[illegible] value of a number of small centres coincides with English experience; a centre for each 1,000 births is approved, though the estimate of the London Women's Co-operative Guild that a clinic could be satisfactorily run for a little under £200 a year is thought to require demonstration.

The chapter on Maternity Benefit also strengthens some conclusions reached in England. One point emphasised is the need of prompt payment. People with ba[illegible] balances do not always remember the great importance [illegible] the poor of receiving money given for a definite purpose at the time when it is needed. Delay may prevent the mother from having the extra food needed, adding a pinch to her other sufferings; the money may ultimately be spent merely in providing clothes for the child. Dr. Sutherland's 1913 inquiry unfortunately went to show that in some cases care was needed to see that the money reached the woman herself, or was expended for her benefit. Sometimes a husband who received it took a holiday, refusing offered jobs, or drank away the whole 30s. Dr. Sutherland's own suggestion is that the money should be paid after the twenty-eighth week of pregnancy, a rule which would certainly involve notification. For those who have leisure to read them the "special studies" are of great interest, bringing forward points of detail that may be highly significant. For instance, the statement of Dr. Saxby, after a lifetime experience in the Shetland island of Unst, is noteworthy, that in the crofting districts, covering the whole island, except the town of Baltasound, labour is as a rule quite normal, but among the town women, whose life is eminently sedentary, some skilled help is nearly always required in consequence of muscular inertia. But the magnificent air and healthy life enjoyed by the crofter child can barely cope with the stomach troubles caused by a diet of boiled tea, salt fish, and undercooked oat-cakes. Cocoa, however, is coming into fashion, and before the sugar scarcity became felt vast masses of sweets were consumed. Dr. Saxby also hazards a guess that the numerous defects of vision noticed in school children here may be due to a habit of violent cradle-rocking, which may "roughly drag away" the eye from the object on which it has fixed. A curious remnant of superstition comes from the neighbouring island of Yell, where a woman testified to having been successfully attended eleven times by a midwife who not only brought a Testament from which to read a chapter at intervals, but also a razor to put under the pillow "for luck."

**CARNEGIE REPORTS ON PHYSICAL WELFARE (4)—(*continued*).**

The great problem of the illegitimate child, of which Scotland offers each year some 8,600 cases, is frankly faced. Dr. Mackenzie owns that longer study means deepening sense of its intricacy. The "angry readiness of father and mother to turn a pregnant unmarried daughter out of the house" is a crime for which he thinks a penal code should contain punishment. But his suggestion of some few remedies includes, besides education in the dangers of drugs and all quackery, two that need insisting on in England: education of boys in the results to women of their self-indulgence, and education "that the good home—father, mother, and child—is the one method of avoiding irreparable injury to the child." In a touching epilogue he adds that if the long pleading of this great volume for better care of mothers and children fails, words are useless, "but perhaps in these days of a common sorrow the cry of every mother's heart for her dead son will keep us to our duty."

